# Supplementary material for: Synthesis and Characterizations of Zinc Oxide on Reduced Graphene Oxide for High Performance Electrocatalytic Reduction of Oxygen
Source: Molecules. 2018 Dec 6;23(12):3227. doi: 10.3390/molecules23123227 (PMC6321287; doi:10.3390/molecules23123227)
Supplement: Supplementary file 1 [file molecules-23-03227-s001.pdf]

Supplementary Information

Synthesis and characterizations of zinc oxide on reduced graphene oxide for high performance electrocatalytic reduction of oxygen

Jiemei Yu[1, 2], Taizhong Huang[2], Zhankun Jiang[2], Min Sun [2*], Chengchun Tang[1*]

1. School of Materials Science and Engineering, Hebei University of Technology, 8 of First Road of Dingzigu, Hongqiao District, Tianjin 300130, China

2. School of Chemistry and Chemical Engineering, University of Jinan, 336 West Nanxinzhuang Road, Jinan 250022, China

Instrumentation and Measurements

The products were characterized by powder X-ray diffraction (XRD, Cu Ka irradiation; λ = 0.154 nm) with a SIEMENS D5000 X-ray diffractometer. The morphology of the synthesized samples was tested by scanning electron microscopy (SEM, JEOL JSM-6701F electron microscope operating at 5 KV). Transmission electron microscopy (TEM) images were examined by a Philips Tecnai 20U-TWIN transmission electron microscope with linear resolution of 0.14 nm and dot resolution of 0.19 nm. Raman spectra tests were conducted by a TriVista™ 555CRS Raman spectrometer at 785 nm. X-ray photoelectron spectroscopy (XPS) data was collected by an ESCALABMKII X-ray photoelectron spectrometer (VG Scienta, USA) equipped with a monochromatic Al Kα X-ray source (1486.6 eV). The pressure in the chamber during the measurements was kept at 1×10^{-7} Pa. The analyzer was operated at a pass energy of 50 eV for high resolution scans and at a pass energy of 100 eV for survey scans. The binding energy of the C 1s peak at 284.6 eV was taken as a reference for the binding energy calibration. A background subtraction and peak fitting were deconvolved using the XPS peak fitting software (XPSPEAK41 by Prof. R. W. M. Kwok).

Electrode preparation and electrochemical tests

5 mg of the prepared catalyst powder was dispersed in the mixture of 450 µL of deionized water and 50 µL of Nafion (5 wt% solution alcohols, DuPont). The mixture was fully sonicated to form a homogeneous ink. Then 5 µL of the ink was dropped onto a glassy carbon (GC) electrode of 3 mm in diameter and fully dried. Cyclic voltammetry measurements were performed using a CHI 760E electrochemical workstation (CH Instrument, USA) by conventional three-electrode cell. The coated glass carbon (GC) electrode is employed as the working electrode, graphite as the counter-electrode, and a saturated calomel electrode (Hg/Hg_2Cl_2) (SCE) as the reference electrode.

Before the ORR tests, cyclic voltammetry (CV) tests were performed from 0.2 to -0.8 V at 5 mV/s in Ar-saturated electrolyte to clean the electrode surface. 20 cycles were carried out to stabilize the current-potential signal. Thereafter, the electrolyte was saturated with oxygen before the start of every experiment by bubbling O_2 at least 30 min, which was maintained over the electrolyte in order to ensure its continued O_2 saturation during the recording. The working electrode was cycled at least 20 cycles before data were recorded at a scan rate of 5 mV/s from 0.2 to -0.8 V vs. Hg/Hg_2Cl_2 in O_2-saturated 0.1 mol/L KOH electrolytes.

The Tafel tests were also conducted at a sweeping rate of 5 mV/s. Rotating disk electrode (RDE) and rotating ring disk electrode (RRDE) tests were performed using a RRDE-3A electrode at the same sweeping rate. For RRDE tests, the working electrode was a glassy carbon disk (5.61 mm in diameter) and a platinum ring leading to a collection efficiency of the ring disk electrode. The RRDE tests were performed at 1600 rpm in O_2-saturated solution. The Pt ring electrode was polarized at -0.3 V vs. Hg/Hg_2Cl_2 for oxidizing the hydrogen peroxide ion during oxygen reduction at the modified GC disk electrode. All the experiments were carried out in 0.1 mol/L KOH solution at room temperature.

The Tafel tests were also conducted at a sweeping rate of 5 mV/s. the exchange current density was derived from the mass-transport correction using Eq. (1):

$$E = E_0 + \frac{2.303RT}{an_aF}\log i_0 - \frac{2.303RT}{an_aF}\log i_d \qquad (1)$$

Where E represents the tested electrode potential, E_0 is the thermodynamics electrode potential, F is the Faraday constant, R is the ideal gas constant, T is the thermodynamic temperature, i_d is the measured current density, and i_0 is the exchange current density.

Rotating disk electrode (RDE) and rotating ring disk electrode (RRDE) tests were performed using a RRDE-3A electrode at the same sweeping rate. For RRDE tests, the working electrode was a glassy carbon disk (5.61 mm in diameter) and a platinum ring leading to a collection efficiency of the ring disk electrode. The RRDE tests were performed at 1600 rpm in O_2-saturated solution. The Pt ring electrode was polarized at -0.3 V vs. Hg/Hg_2Cl_2 for oxidizing the hydrogen peroxide ion during oxygen reduction at the modified GC disk electrode. All the experiments were carried out in 0.1 M KOH solution at room temperature.

For RDE test ,the transferred electron numbers of oxygen reduction could be calculated according to the following *Koutechy–Levich* equation (Kakaei and Hasanpour, 2014):

$$\frac{1}{I} = \frac{1}{I_k} + \frac{1}{Bw^{0.5}} \qquad (2)$$

Where I_k is the kinetic current and w is the angular velocity (w = 2πN, N is the linear rotation speed). B could be determined from the slope of the K–L plots based on the *Koutechy–Levich* equation as follows:

$$B = 0.62nF(D_{O_2})^{2/3}v^{-1/6}C_{O_2} \qquad (3)$$

Where n represents the transferred electron number, F is the Faraday constant (F = 96485 C/mol), D_{O_2} is the diffusion coefficient of O_2 in 0.1 M KOH (1.9×10^{-5} cm^2/s), v is the kinetic viscosity (0.01 cm^2/s), and C_{O_2} is the bulk concentration of O_2 (1.2×10^{-7} mol/cm^3).

For RRDE test, the ORR percentage of peroxide species and electron transfer numbers with respect to total ORR products on CuO/rGO were calculated from the following equations:

$$\%H_2O_2 = 200 \times \frac{I_r / N}{I_d + I_r / N} \qquad (4)$$

$$n = 4 \times \frac{I_d}{I_d + I_r / N} \qquad (5)$$

Where I_d is the disk current, I_r is the ring current, and N is the current collection efficiency of the Pt ring 0.39.
